# Supplementary material for: Exploring the cocrystallization potential of urea and benzamide
Source: J Mol Model. 2016 Apr 6;22:103. doi: 10.1007/s00894-016-2964-6 (PMC4823316; doi:10.1007/s00894-016-2964-6)
Supplement: Supplementary file 1 — (DOCX 11435 kb) [file 894_2016_2964_MOESM1_ESM.docx]

**Supporting materials**

**Exploring cocrystallization potential of urea and benzamide**

Piotr Cysewski^1*^, Maciej Przybyłek^1^, Dorota Ziółkowska^2^ and Karina Mroczyńska^3^

^1^Chair and Department of Physical Chemistry, Collegium Medicum of Bydgoszcz, Nicolaus Copernicus University in Toruń, Kurpińskiego 5, 85-950 Bydgoszcz, Poland, piotr.cysewski@cm.umk.pl

^2^University of Technology and Life Sciences in Bydgoszcz, Faculty of Chemical Technology and Engineering, Seminaryjna 3, 85-326 Bydgoszcz, Poland;

^4^Research Laboratory, Faculty of Chemical Technology and Engineering, Seminaryjna 3, 85-326 Bydgoszcz

**I. Experimental evidences of urea cocrystallization**

**Fig. S1** The recorded PXRD and ATR-FTIR spectra of urea (U), oxalic acid (OA) and U-OA mixture crystallites deposited on glass surfaces and simulated powder patterns calculated using CIF deposited in CSD (Cambridge Structural Database) [30].

**Fig. S2** The recorded PXRD and ATR-FTIR spectra of urea (U), malonic acid (MOA) and U-MOA mixture crystallites deposited on glass surfaces and simulated powder patterns calculated using CIF deposited in CSD (Cambridge Structural Database) [30].

**Fig. S3** The recorded PXRD and ATR-FTIR spectra of urea (U), maleic acid (MEA) and U-MEA mixture crystallites deposited on glass surfaces and simulated powder patterns calculated using CIF deposited in CSD (Cambridge Structural Database) [30].

**Fig. S4** The recorded PXRD and ATR-FTIR spectra of urea (U), fumaric acid (FA) and U-FA mixture crystallites deposited on glass surfaces and simulated powder patterns calculated using CIF deposited in CSD (Cambridge Structural Database) [30].

**Fig. S5** The recorded PXRD and ATR-FTIR spectra of urea (U), succinic acid (SUA) and U-SUA mixture crystallites deposited on glass surfaces a and simulated powder patterns calculated using CIF deposited in CSD (Cambridge Structural Database) [30].

**Fig. S6** The recorded PXRD and ATR-FTIR spectra of urea (U), glutaric acid (GA) and U-GA mixture crystallites deposited on glass surfaces and simulated powder patterns calculated using CIF deposited in CSD (Cambridge Structural Database) [30].

**II. Experimental evidences of benzamide cocrystallization**

**Fig. S7** The recorded PXRD and ATR-FTIR spectra of benzamide (B), oxalic acid (OA) and B-OA mixture crystallites deposited on glass surfaces and simulated powder patterns calculated using CIF deposited in CSD (Cambridge Structural Database) [30].

**Fig. S8** The recorded PXRD and ATR-FTIR spectra of benzamide (B), malonic acid (MOA) and B-MOA mixture crystallites deposited on glass surfaces and simulated powder patterns calculated using CIF deposited in CSD (Cambridge Structural Database) [30].

**Fig. S9** The recorded PXRD and ATR-FTIR spectra of benzamide (B), maleic acid (MEA) and B-MEA mixture crystallites deposited on glass surfaces and simulated powder patterns calculated using CIF deposited in CSD (Cambridge Structural Database) [30].

**Fig. S10** The recorded PXRD and ATR-FTIR spectra of benzamide (B), fumaric acid (FA) and B-FA mixture crystallites deposited on glass surfaces and simulated powder patterns calculated using CIF deposited in CSD (Cambridge Structural Database) [30].

**Fig. S11** The recorded PXRD and ATR-FTIR spectra of benzamide (B), succinic acid (SUA) and B-SUA mixture crystallites deposited on glass surfaces and simulated powder patterns calculated using CIF deposited in CSD (Cambridge Structural Database) [30].

**Fig. S12** The recorded PXRD and ATR-FTIR spectra of benzamide (B), glutaric acid (GA) and B-GA mixture crystallites deposited on glass surfaces and simulated powder patterns calculated using CIF deposited in CSD (Cambridge Structural Database) [30].

**Fig. S13** The recorded PXRD and ATR-FTIR spectra of benzamide (B), salicylic acid (SA) and B-SA mixture crystallites deposited on glass surfaces and simulated powder patterns calculated using CIF deposited in CSD (Cambridge Structural Database) [30].

**Fig. S14** The recorded PXRD and ATR-FTIR spectra of benzamide (B), 2,5-dihydroxybenzoic acid (2,5DHBA) and B-2,5DHBA mixture crystallites deposited on glass surfaces and simulated powder patterns calculated using CIF deposited in CSD (Cambridge Structural Database) [30].

**Fig. S15** The recorded PXRD and ATR-FTIR spectra of benzamide (B), 2,6-dihydroxybenzoic acid (2,6DHBA) and B-2,6DHBA mixture crystallites deposited on glass surfaces and simulated powder patterns calculated using CIF deposited in CSD (Cambridge Structural Database) [30].

**Fig. S16** The recorded PXRD and ATR-FTIR spectra of benzamide (B), 3,5-dihydroxybenzoic acid (3,5DHBA) and B-3,5DHBA mixture crystallites deposited on glass surfaces and simulated powder patterns calculated using CIF deposited in CSD (Cambridge Structural Database) [30].

**III. Experimental evidences of benzamide immiscibility in solid phase**

**Fig. S17** The recorded PXRD and ATR-FTIR spectra of benzamide (B), benzoic acid (BA) and B-BA mixture crystallites deposited on glass surfaces and simulated powder patterns calculated using CIF deposited in CSD (Cambridge Structural Database) [30].

**Fig. S18** The recorded PXRD and ATR-FTIR spectra of benzamide (B), 3-hydroxybenzoic acid (3HBA) and B-3HBA mixture crystallites deposited on glass surfaces and simulated powder patterns calculated using CIF deposited in CSD (Cambridge Structural Database) [30].

**Fig. S19** The recorded PXRD and ATR-FTIR spectra of benzamide (B), 4-hydroxybenzoic acid (4HBA) and B-4HBA mixture crystallites deposited on glass surfaces and simulated powder patterns calculated using CIF deposited in CSD (Cambridge Structural Database) [30].

**IV. Details of COSMOtherm computations**

**Table S1**. The list of pairs of benzamide and urea binary mixtures with carboxylic acids. The values of excess thermodynamic functions are expressed in kcal/mol.

|  | **benzamide** | | **urea** | |
| --- | --- | --- | --- | --- |
| **carboxylic acid** | H^mix^ | G^mix^ | H^mix^ | G^mix^ |
| lactic acid | -0.256 | -0.623 | -0.236 | -0.670 |
| ascorbic acid | -0.272 | -0.676 | -0.549 | -0.962 |
| 4-methylsalicylic acid | -0.227 | -0.787 | 0.076 | -0.583 |
| 1-amino-cyclopentanecarboxylic acid | -1.093 | -0.559 | -1.039 | -0.704 |
| isonicotinic acid | -0.066 | -0.483 | 0.245 | -0.316 |
| 4-aminobutanoic acid | 3.104 | 0.651 | 1.177 | 0.045 |
| aspartic acid | 0.176 | 0.000 | -0.420 | -0.687 |
| glutamic acid | 0.244 | -0.060 | -0.102 | -0.545 |
| hexadecanoic acid | 0.364 | -0.363 | 1.047 | 0.140 |
| octadecanoic acid | 0.394 | -0.339 | 1.108 | 0.189 |
| 5,5-diethylbarbituric acid | -0.155 | -0.551 | 0.002 | -0.471 |
| 3-pyridinecarboxylic acid | -0.076 | -0.483 | 0.208 | -0.329 |
| ethylenediaminetetraacetic acid | -0.076 | -0.612 | -0.212 | -0.864 |
| aminocaproic acid | 0.394 | 0.191 | 0.439 | -0.196 |
| (z,z)-9,12-octadecadienoic acid | 0.268 | -0.422 | 0.971 | 0.072 |
| 4-nitrobenzoic acid | -0.345 | -0.897 | -0.276 | -0.839 |
| alpha-aminoisobutanoic acid | -0.513 | -0.316 | -0.626 | -0.636 |
| formic acid | -0.253 | -0.816 | -0.541 | -1.048 |
| acetic acid | -0.183 | -0.649 | -0.201 | -0.706 |
| iodoacetic acid | -0.436 | -0.964 | -0.411 | -0.946 |
| benzoic acid | -0.153 | -0.712 | 0.055 | -0.562 |
| orotic acid | -0.272 | -0.657 | -0.578 | -0.961 |
| barbituric acid | 0.038 | -0.396 | -0.383 | -0.736 |
| mercaptoacetic acid | -0.379 | -0.725 | -0.555 | -0.812 |
| salicylic acid | -0.329 | -0.856 | -0.104 | -0.705 |
| alpha-aminobenzeneacetic acid | -0.093 | -0.297 | -0.044 | -0.487 |
| mercapto-butanedioic acid | -0.558 | -0.875 | -0.849 | -1.086 |
| 4-chlorobenzoic acid | -0.231 | -0.775 | -0.015 | -0.605 |
| isocyanic acid | -0.963 | -0.923 | -0.952 | -0.837 |
| cacodylic acid | -0.217 | -0.330 | -0.255 | -0.360 |
| methanesulfonic acid | -0.563 | -1.032 | -1.043 | -1.381 |
| 2,2-dimethyl-propanoic acid | -0.033 | -0.588 | 0.234 | -0.391 |
| trichloroacetic acid | -1.229 | -1.540 | -1.184 | -1.475 |
| chlorodifluoroacetic acid | -1.171 | -1.580 | -1.130 | -1.533 |
| trifluoroacetic acid | -1.008 | -1.486 | -0.981 | -1.452 |
| 2-hydroxy-1,2,3-propanetricarboxylic acid | -0.401 | -0.886 | -0.690 | -1.215 |
| bromoacetic acid | -0.576 | -0.973 | -0.637 | -1.007 |
| propionic acid | -0.161 | -0.666 | -0.073 | -0.618 |
| acrylic acid | -0.221 | -0.701 | -0.166 | -0.695 |
| chloroacetic acid | -0.527 | -0.930 | -0.717 | -1.057 |
| hydroxy-acetic acid | -0.178 | -0.637 | -0.483 | -0.866 |
| peroxyacetic acid | 0.001 | -0.407 | -0.052 | -0.296 |
| isobutyric acid | -0.114 | -0.642 | 0.106 | -0.510 |
| methacrylic acid | -0.140 | -0.669 | 0.010 | -0.567 |
| thiolactic acid | -0.426 | -0.777 | -0.424 | -0.742 |
| dichloroacetic acid | -0.927 | -1.314 | -0.997 | -1.337 |
| tetrahydro-2,2-dimethyl-3-furancarboxylic acid | -0.120 | -0.621 | -0.006 | -0.596 |
| a-bromobutyric acid | -0.446 | -0.928 | -0.217 | -0.773 |
| tiglic acid | -0.090 | -0.589 | 0.153 | -0.419 |
| 2-amino-butyric acid | 0.088 | -0.099 | 0.029 | -0.417 |
| tartronic acid | -0.738 | -0.954 | -1.297 | -1.291 |
| 3-methylsalicylic acid | -0.266 | -0.835 | 0.082 | -0.608 |
| anthraflavic acid | -0.426 | -1.018 | -0.280 | -0.994 |
| 1-naphthalenecarboxylic acid | -0.127 | -0.675 | 0.265 | -0.421 |
| indoleacetic acid | -0.467 | -0.764 | -0.344 | -0.665 |
| 2-ethylbutyric acid | 0.018 | -0.564 | 0.419 | -0.310 |
| 3-thenoic acid | -0.208 | -0.732 | -0.071 | -0.630 |
| 2-furancarboxylic acid | -0.277 | -0.737 | -0.294 | -0.739 |
| 2-methylbenzenesulfonic acid | -0.634 | -1.170 | -0.534 | -1.114 |
| 2-bromobenzoic acid | -0.338 | -0.744 | -0.111 | -0.562 |
| 2-iodobenzoic acid | -0.277 | -0.777 | -0.013 | -0.576 |
| phthalic acid | -0.050 | -0.659 | 0.043 | -0.613 |
| quinolinic acid | -0.129 | -0.394 | -0.099 | -0.249 |
| 1,2,4,5-benzenetetracarboxylic acid | -0.976 | -1.308 | -1.216 | -1.683 |
| 5-methylsalicylic acid | -0.242 | -0.796 | 0.052 | -0.591 |
| 5-amino-2-hydroxybenzoic acid | -0.227 | -0.645 | -0.062 | -0.572 |
| salicylhydroxamic acid | -0.173 | -0.704 | -0.043 | -0.564 |
| 2,4-dihydroxybenzoic acid | -0.588 | -1.076 | -0.504 | -1.115 |
| a-ethylbenzeneacetic acid | -0.099 | -0.665 | 0.291 | -0.393 |
| mandelic acid | -0.214 | -0.674 | -0.067 | -0.580 |
| 2,4-dimethoxybenzoic acid | -0.225 | -0.423 | 0.058 | -0.137 |
| 3-hydroxy-2-naphthoic acid | -0.242 | -0.850 | 0.090 | -0.623 |
| 4-phenylbenzoic acid | -0.070 | -0.657 | 0.339 | -0.372 |
| 3,4-dimethoxybenzoic acid | -0.086 | -0.623 | 0.171 | -0.439 |
| 2-naphthalenecarboxylic acid | -0.120 | -0.692 | 0.212 | -0.454 |
| 2-methoxybenzeneacetic acid | -0.284 | -0.570 | -0.117 | -0.396 |
| perbenzoic acid | -0.333 | -0.963 | -0.189 | -0.872 |
| (2,4,5-trichlorophenoxy)-acetic acid | -0.625 | -0.908 | -0.479 | -0.758 |
| 1,3-benzodioxole-5-carboxylic acid | -0.208 | -0.690 | -0.043 | -0.575 |
| 2-methylvaleric acid | 0.001 | -0.559 | 0.375 | -0.305 |
| methylenebutanedioic acid | -0.222 | -0.712 | -0.335 | -0.824 |
| malic acid | -0.307 | -0.722 | -0.529 | -0.917 |
| phenylarsonic acid | -0.183 | -0.678 | -0.213 | -0.755 |
| benzenesulfonic acid | -0.744 | -1.219 | -0.749 | -1.231 |
| p-chlorobenzenesulfonic acid | -0.820 | -1.300 | -0.815 | -1.283 |
| 4-(1,1-dimethylethyl)-benzoic acid | 0.002 | -0.599 | 0.445 | -0.289 |
| phenylboronic acid | -0.191 | -0.683 | -0.024 | -0.564 |
| cyclohexanecarboxylic acid | -0.031 | -0.573 | 0.331 | -0.315 |
| benzenecarbothioic acid | 0.116 | -0.303 | 0.416 | 0.067 |
| pyrazinoic acid | -0.316 | -0.462 | -0.292 | -0.343 |
| picolinic acid | -0.040 | -0.364 | 0.109 | -0.145 |
| 3-methylbenzoic acid | -0.101 | -0.672 | 0.193 | -0.463 |
| 3-aminobenzoic acid | -0.192 | -0.677 | -0.116 | -0.624 |
| 3-hydroxybenzoic acid | -0.570 | -0.976 | -0.523 | -1.019 |
| 3,5-dihydroxybenzoic acid | -0.710 | -1.082 | -0.887 | -1.309 |
| 3,5-dinitrobenzoic acid | -0.624 | -1.108 | -0.639 | -1.113 |
| 3,4-dihydroxybenzoic acid | -0.799 | -1.093 | -0.923 | -1.223 |
| valproic acid | 0.115 | -0.503 | 0.577 | -0.175 |
| 4-methyl-benzoic acid | -0.106 | -0.656 | 0.179 | -0.451 |
| 4-oh-benzoic acid | -0.673 | -1.021 | -0.640 | -1.068 |
| 4-methoxybenzoic acid | -0.130 | -0.623 | 0.110 | -0.455 |
| 1,4-benzenedicarboxylic acid | -0.509 | -1.001 | -0.479 | -1.063 |
| pyridine-2,5-dicarboxylic acid | -0.155 | -0.724 | -0.154 | -0.734 |
| 2-(m-chlorophenoxy)propionic acid | -0.525 | -0.824 | -0.323 | -0.648 |
| phenylacetic acid | -0.196 | -0.720 | 0.045 | -0.567 |
| 4-methoxy-benzeneacetic acid | -0.199 | -0.673 | 0.095 | -0.498 |
| 4-methylbenzenesulfonic acid | -0.594 | -1.086 | -0.518 | -1.041 |
| 3-(1h-imidazol-4-yl)-2-propenoic acid | -0.008 | -0.462 | 0.041 | -0.531 |
| 3-methylpentanoic- acid | -0.025 | -0.570 | 0.336 | -0.328 |
| 2-(ethylamino)-ethanesulfonic acid | 0.677 | 0.054 | -0.003 | -0.506 |
| butyric acid | -0.108 | -0.629 | 0.117 | -0.497 |
| trans-crotonic- acid | -0.142 | -0.618 | 0.046 | -0.522 |
| 3-chloropropanoic acid | -0.290 | -0.816 | -0.347 | -0.856 |
| 3-mercapto-propanoic acid | -0.274 | -0.759 | -0.247 | -0.750 |
| succinic acid | -0.258 | -0.692 | -0.496 | -0.919 |
| (z)-2-butenedioic acid | -0.125 | -0.741 | -0.244 | -0.866 |
| fumaric acid | -0.623 | -1.077 | -0.807 | -1.318 |
| sorbinic acid | -0.063 | -0.574 | 0.186 | -0.407 |
| pentanedioic acid | -0.394 | -0.728 | -0.527 | -0.874 |
| 2,2'-oxybisacetic acid | -0.148 | -0.693 | -0.364 | -0.891 |
| n-heptanoic acid | 0.038 | -0.566 | 0.427 | -0.279 |
| heptanedioic acid | -0.293 | -0.735 | -0.149 | -0.745 |
| thiodipropionic- acid | -0.508 | -0.742 | -0.603 | -0.837 |
| decanedioic acid | -0.197 | -0.710 | 0.104 | -0.572 |
| nonanoic acid | 0.109 | -0.505 | 0.596 | -0.147 |
| undecanoic acid | 0.185 | -0.451 | 0.785 | -0.029 |
| 10-undecenoic- acid | 0.079 | -0.517 | 0.637 | -0.122 |
| cis-9-octadecenoic acid | 0.299 | -0.380 | 1.005 | 0.133 |
| docosanoic acid | 0.509 | -0.263 | 1.331 | 0.317 |
| erucic- acid | 0.417 | -0.328 | 1.192 | 0.231 |
| 2-methylbutanoic acid | -0.044 | -0.601 | 0.251 | -0.404 |
| 2-hydroxyphenylesterbenzoic acid | 0.260 | -0.217 | 0.758 | 0.269 |
| 2-methyl-benzoic acid | -0.110 | -0.653 | 0.209 | -0.423 |
| 2-chloro-benzoic acid | -0.356 | -0.742 | -0.176 | -0.603 |
| 2-aminobenzoic acid | -0.340 | -0.730 | -0.144 | -0.577 |
| 5-iodo-2-hydroxybenzoic acid | -0.411 | -1.010 | -0.115 | -0.783 |
| 2-formylbenzoic acid | -0.133 | -0.450 | -0.049 | -0.273 |
| 2-(methylamino)benzoic acid | -0.101 | -0.634 | 0.290 | -0.370 |
| vanillic- acid | -0.382 | -0.764 | -0.305 | -0.689 |
| 4-aminobenzenesulfonic acid | 0.080 | -0.165 | -0.191 | -0.875 |
| 1,3-benzenedicarboxylic acid | -0.442 | -0.955 | -0.391 | -1.015 |
| 3-nitrobenzoic acid | -0.343 | -0.876 | -0.248 | -0.815 |
| triethylesterphosphorous acid | 0.234 | -0.170 | 0.846 | 0.396 |
| phenoxyacetic acid | -0.500 | -0.781 | -0.391 | -0.678 |
| sulfoacetic acid | -1.023 | -1.342 | -1.479 | -1.682 |
| 4-oxo-pentanoic acid | -0.255 | -0.503 | -0.215 | -0.484 |
| 2,2'-thiobisacetic acid | -0.126 | -0.732 | -0.277 | -0.870 |
| nonanedioic acid | -0.220 | -0.729 | 0.010 | -0.644 |
| hexanedioic acid | -0.380 | -0.734 | -0.412 | -0.804 |
| caprylic acid | 0.071 | -0.529 | 0.559 | -0.194 |
| methylarsonic acid | -0.046 | -0.425 | -0.411 | -0.805 |
| pyruvic acid | -0.470 | -0.640 | -0.701 | -0.707 |
| pimaric acid | 0.168 | -0.473 | 0.830 | -0.004 |
| 2,4,6-trinitrobenzoic acid | -0.995 | -1.334 | -1.109 | -1.414 |
| pamoic acid | -0.479 | -1.086 | -0.062 | -0.855 |
| tartaric acid | -0.425 | -0.831 | -0.837 | -1.204 |
| 2-ethoxybenzoic acid | -0.164 | -0.398 | 0.186 | -0.067 |
| 4-sulfamoylbenzoic acid | -0.327 | -0.744 | -0.740 | -0.966 |
| 4-nitrobenzenesulfonic acid | -0.839 | -1.372 | -0.932 | -1.433 |
| cinnamic acid | -0.080 | -0.625 | 0.199 | -0.427 |
| propanedioic acid | -0.156 | -0.749 | -0.469 | -1.004 |
| n-hexanoic acid | -0.012 | -0.573 | 0.325 | -0.328 |
| iminodiacetic acid | 0.030 | -0.482 | -0.245 | -0.721 |
| dodecanoic acid | 0.227 | -0.440 | 0.829 | -0.003 |
| 2-fluoroacetic acid | -0.407 | -0.802 | -0.636 | -0.970 |
| oxalic acid | -1.153 | -1.268 | -1.731 | -1.622 |
| 2-mercaptobenzoic acid | -0.329 | -0.851 | -0.135 | -0.686 |
| 2-ethyl-hexanoic acid | 0.122 | -0.502 | 0.590 | -0.174 |
| 3,4,5-trihydroxybenzoic acid | -0.915 | -1.138 | -1.142 | -1.337 |
| 4-aminobenzoic acid | -0.237 | -0.642 | -0.215 | -0.607 |
| 2-oxo-3-phenylpropionic- acid | -0.395 | -0.589 | -0.361 | -0.435 |
| p-hydroxyphenylacetic acid | -0.435 | -0.869 | -0.386 | -0.916 |
| glyoxylic acid | -0.728 | -0.977 | -1.085 | -1.184 |
| 3-hydroxybutyric acid | -0.174 | -0.500 | -0.076 | -0.485 |
| 2,6-dihydroxybenzoic acid | -0.227 | -0.908 | -0.080 | -0.833 |
| 2,3-dihydroxybenzoic acid | -0.482 | -0.977 | -0.429 | -0.921 |
| oxalacetic acid | -0.649 | -0.976 | -1.111 | -1.291 |
| 2-oxopentanedioic acid | -0.501 | -0.851 | -0.816 | -1.097 |
| m-fluorophenylacetic acid | -0.234 | -0.757 | -0.002 | -0.602 |
| decanoic acid | 0.149 | -0.473 | 0.715 | -0.075 |
| (e)-2-decenoic- acid | 0.142 | -0.466 | 0.706 | -0.081 |
| cyanoacetic acid | -0.167 | -0.808 | -0.449 | -1.027 |
| perfluorobutanoic acid | -0.948 | -1.438 | -0.764 | -1.275 |
| tridecafluoroheptanoic acid | -0.802 | -1.265 | -0.492 | -0.992 |
| difluoroacetic acid | -0.777 | -1.244 | -0.895 | -1.326 |
| 2,6-difluorobenzoic acid | -0.412 | -0.812 | -0.264 | -0.697 |
| p-fluorophenylacetic acid | -0.214 | -0.742 | 0.026 | -0.586 |
| cyanic acid | -0.556 | -1.410 | -0.765 | -1.613 |
| 4-thiazolidinecarboxylic acid | -0.215 | -0.461 | -0.228 | -0.399 |
| 2-fluorobenzoic acid | -0.340 | -0.714 | -0.185 | -0.572 |
| o-fluorophenylacetic acid | -0.200 | -0.729 | 0.050 | -0.578 |
| 4-trifluoromethylbenzoic acid | -0.204 | -0.801 | 0.126 | -0.569 |
| 3-fluorobenzoic acid | -0.213 | -0.767 | 0.027 | -0.601 |
| 4-fluorobenzoic acid | -0.187 | -0.733 | 0.040 | -0.567 |
| 9,12,15-octadecatrienoic acid | 0.248 | -0.448 | 0.929 | 0.040 |
| thiocyanic acid | -0.213 | -0.496 | -0.259 | -0.401 |
| propargylic acid | -0.650 | -1.070 | -0.768 | -1.165 |
| chlorofluoroacetic acid | -0.912 | -1.279 | -0.999 | -1.335 |
| oxamic acid | -0.079 | -0.485 | -0.520 | -0.744 |
| dehydro(2)abietic acid | 0.173 | -0.480 | 0.859 | 0.001 |
| glyceric acid | -0.169 | -0.564 | -0.389 | -0.768 |
| 2,4,6-trimethylbenzoic acid | -0.027 | -0.621 | 0.389 | -0.329 |
| [1,1'-biphenyl]-2,2'-dicarboxylic acid | 0.010 | -0.654 | 0.293 | -0.499 |
| 3-furancarboxylic acid | -0.275 | -0.769 | -0.251 | -0.739 |
| cinchomeronic acid | -0.175 | -0.590 | -0.126 | -0.603 |
| 2,5-dihydroxybenzoic acid | -0.429 | -0.956 | -0.364 | -1.002 |
| atropic acid | -0.196 | -0.711 | 0.081 | -0.516 |
| benzohydroxamic acid | -0.086 | -0.508 | 0.155 | -0.331 |
| 2-benzofurancarboxylic acid | -0.285 | -0.757 | -0.087 | -0.615 |
| 2-amino-pentanoic acid | -0.109 | -0.240 | -0.010 | -0.418 |
| pyrotartaric acid | -0.242 | -0.678 | -0.327 | -0.781 |
| citraconic acid | -0.085 | -0.684 | -0.059 | -0.705 |
| (e)-2-methyl-2-butenedioic acid | -0.521 | -0.986 | -0.521 | -1.106 |
| isonipecotic acid | -0.417 | -0.287 | -0.280 | -0.220 |
| nipecotic acid | -0.158 | -0.124 | 0.071 | -0.027 |
| 1,2,5,6-tetrahydro-1-methylnicotinic acid | -0.055 | -0.404 | 0.383 | -0.123 |
| 3,5-dimethyl-benzoic acid | -0.051 | -0.632 | 0.318 | -0.371 |
| 2,4-pyridinedicarboxylic acid | -0.130 | -0.722 | -0.120 | -0.742 |
| dinicotinic acid | -0.248 | -0.767 | -0.147 | -0.834 |
| pyridine-2,6-dicarboxylic acid | -0.312 | -0.651 | -0.333 | -0.680 |
| 2-oxo-2h-pyran-5-carboxylic acid | -0.260 | -0.755 | -0.305 | -0.799 |
| oxo(phenylamino)acetic acid | -0.119 | -0.471 | -0.002 | -0.271 |
| beta-phenylpropionic acid | -0.155 | -0.703 | 0.157 | -0.489 |
| 3-pyridineacetic acid | -0.043 | -0.430 | 0.261 | -0.270 |
| p-hydroxy-b-propionic acid | -0.390 | -0.844 | -0.256 | -0.825 |
| 3,7-dimethyl-6-octenoic- acid | 0.049 | -0.545 | 0.545 | -0.197 |
| (z)-2-butenoic acid | -0.131 | -0.637 | 0.045 | -0.531 |
| hydracrylic acid | -0.163 | -0.498 | -0.293 | -0.627 |
| isovaleric acid | -0.067 | -0.610 | 0.231 | -0.415 |
| octanedioic acid | -0.271 | -0.728 | -0.094 | -0.680 |
| hexadecanedioic acid | -0.016 | -0.629 | 0.527 | -0.316 |
| heptadecanoic acid | 0.393 | -0.335 | 1.155 | 0.196 |
| eicosanoic acid | 0.448 | -0.311 | 1.230 | 0.246 |
| thioacetic acid | -0.003 | -0.402 | 0.096 | -0.186 |
| diethylpropanedioic acid | 0.016 | -0.672 | 0.272 | -0.534 |
| abietic acid | 0.179 | -0.445 | 0.879 | 0.048 |
| a-hydroxy-a-methylbenzeneacetic acid | -0.170 | -0.650 | 0.174 | -0.453 |
| methylmalonic acid | -0.225 | -0.766 | -0.357 | -0.888 |
| 2,3-dihydroxybutanedioic acid | -0.483 | -0.863 | -0.886 | -1.235 |
| 2-thiophenecarboxylic acid | -0.237 | -0.750 | -0.071 | -0.642 |
| 1,2,4-benzenetricarboxylic acid | -0.502 | -1.010 | -0.557 | -1.150 |
| 3,4-dinitrobenzoic acid | -0.659 | -1.123 | -0.681 | -1.132 |
| 2,4,5-trimethyl-benzoic acid | 0.008 | -0.566 | 0.431 | -0.260 |
| tropic acid | -0.181 | -0.594 | -0.031 | -0.488 |
| butylmalonic acid | -0.093 | -0.726 | 0.092 | -0.617 |
| 2-piperidinecarboxylic acid | 0.088 | -0.380 | 0.385 | -0.129 |
| 3-chlorobenzoic acid | -0.266 | -0.832 | -0.049 | -0.665 |
| 3,5-diaminobenzoic acid | -0.109 | -0.543 | -0.095 | -0.570 |
| p-toluenesulfinic acid | -0.129 | -0.629 | 0.137 | -0.452 |
| 4-(1-methylethyl)-benzoic acid | -0.019 | -0.616 | 0.380 | -0.331 |
| 3-(2-furanyl)-2-propenoic acid | -0.135 | -0.619 | 0.064 | -0.491 |
| 3-methyl-2-butenoic acid | -0.084 | -0.572 | 0.182 | -0.402 |
| 3-aminobutanoic acid | 0.016 | -0.094 | 0.037 | -0.371 |
| acetoacetic acid | -0.073 | -0.406 | -0.158 | -0.416 |
| 3-oxopentanedioic acid | -0.173 | -0.614 | -0.603 | -0.891 |
| tetradecanoic acid | 0.290 | -0.406 | 0.975 | 0.076 |
| acetohydroxamic acid | 0.006 | -0.423 | -0.114 | -0.499 |
| 2-nitrobenzoic acid | -0.397 | -0.883 | -0.418 | -0.900 |
| 1,3,5-benzenetricarboxylic acid | -0.705 | -1.155 | -0.777 | -1.361 |
| maleamic acid | -0.085 | -0.485 | -0.354 | -0.730 |
| 2,3-dichloropropionic acid | -0.708 | -1.121 | -0.747 | -1.142 |
| 1h-imidazole-4,5-dicarboxylic acid | -0.043 | -0.594 | -0.262 | -0.794 |
| o-acetylbenzoic acid | -0.179 | -0.483 | -0.049 | -0.323 |
| 2-methoxybenzoic acid | -0.137 | -0.401 | 0.116 | -0.139 |
| 4-(methylthio)-2-oxobutanoic- acid | -0.472 | -0.648 | -0.539 | -0.582 |
| 3-bromobenzoic acid | -0.233 | -0.811 | 0.038 | -0.601 |
| 3-methoxybenzoic acid | -0.179 | -0.697 | 0.102 | -0.510 |
| 4-bromobenzoic acid | -0.228 | -0.757 | 0.053 | -0.541 |
| p-acetylbenzoic acid | -0.073 | -0.608 | 0.204 | -0.450 |
| 3-bromopropanoic acid | -0.340 | -0.868 | -0.255 | -0.815 |
| 2-butynoic acid | -0.171 | -0.752 | -0.150 | -0.766 |
| 4-pentenoic acid | -0.183 | -0.681 | 0.015 | -0.551 |
| trithiocarbonic acid | -0.247 | -0.520 | -0.156 | -0.240 |
| ethanesulfonic acid | -0.567 | -1.033 | -0.814 | -1.230 |
| 2-hydroxy-2-methylpropanoic acid | -0.187 | -0.591 | -0.046 | -0.530 |
| 2,2-dimethylbutyric acid | 0.016 | -0.554 | 0.345 | -0.314 |
| dimethylmalonic acid | -0.317 | -0.781 | -0.372 | -0.830 |
| 2,2-dimethylbutanedioic acid | -0.077 | -0.634 | 0.010 | -0.618 |
| a-bromopropionic acid | -0.463 | -0.946 | -0.360 | -0.874 |
| 2-chloropropionic acid | -0.512 | -0.914 | -0.544 | -0.926 |
| 2-chloro-2-butenoic acid | -0.458 | -0.804 | -0.382 | -0.722 |
| 2-hydroxy-butyric acid | -0.173 | -0.615 | -0.052 | -0.567 |
| 2-oxobutanoic acid | -0.361 | -0.585 | -0.469 | -0.535 |
| ethylmalonic acid | -0.180 | -0.753 | -0.176 | -0.781 |
| pentafluorobenzoic acid | -0.425 | -0.964 | -0.209 | -0.783 |
| 2,3-dimethylbenzoic acid | -0.080 | -0.640 | 0.284 | -0.383 |
| 2-(dimethylamino)benzoic acid | -0.657 | -0.521 | -0.285 | -0.229 |
| 2,4-dinitrobenzoic acid | -0.703 | -1.148 | -0.822 | -1.215 |
| 2,5-dimethylbenzoic acid | -0.050 | -0.603 | 0.347 | -0.319 |
| 2,4-dimethylbenzoic acid | -0.036 | -0.587 | 0.352 | -0.309 |
| 2,4-diaminobenzoic acid | -0.237 | -0.630 | -0.221 | -0.601 |
| benzoylformic acid | -0.630 | -0.595 | -0.674 | -0.456 |
| 2-ethylbenzoic acid | -0.084 | -0.645 | 0.322 | -0.369 |
| 3-amino-3-phenylpropanoic acid | -0.086 | -0.427 | 0.045 | -0.250 |
| o-hydroxyphenylacetic acid | -0.149 | -0.785 | 0.045 | -0.672 |
| 2-hydroxypentanoic acid | -0.128 | -0.578 | 0.140 | -0.445 |
| benzenesulfinic acid | -0.160 | -0.683 | -0.006 | -0.576 |
| 3,4-dimethylbenzoic acid | -0.051 | -0.614 | 0.316 | -0.361 |
| 3,4-diaminobenzoic acid | -0.038 | -0.515 | 0.006 | -0.516 |
| 3-ethylbenzoic acid | -0.070 | -0.655 | 0.298 | -0.396 |
| 3-formylbenzoic acid | -0.147 | -0.670 | 0.029 | -0.564 |
| 4-iodobenzoic acid | -0.197 | -0.764 | 0.107 | -0.531 |
| 4-ethylbenzoic acid | -0.058 | -0.615 | 0.334 | -0.353 |
| 4-cyanobenzoic acid | -0.161 | -0.739 | -0.013 | -0.626 |
| trans-cyclohexane-1,4-dicarboxylic acid | -0.311 | -0.779 | -0.189 | -0.791 |
| 4-dimethylaminobenzoic acid | -0.002 | -0.515 | 0.313 | -0.294 |
| p-ethoxybenzoic acid | -0.083 | -0.606 | 0.218 | -0.385 |
| m-methylphenylacetic acid | -0.170 | -0.695 | 0.130 | -0.487 |
| m-hydroxyphenylacetic acid | -0.447 | -0.900 | -0.416 | -0.950 |
| 3-ethoxybenzoic acid | -0.097 | -0.678 | 0.243 | -0.438 |
| (z)-3-phenyl-2-propenoic acid | -0.127 | -0.652 | 0.238 | -0.416 |
| p-methylphenylacetic acid | -0.186 | -0.694 | 0.103 | -0.489 |
| 5-oxodecanoic- acid | -0.254 | -0.495 | 0.164 | -0.171 |
| 3-hydroxy-3-methylbutanoic acid | -0.192 | -0.494 | 0.050 | -0.378 |
| 3-butenoic acid | -0.198 | -0.699 | -0.105 | -0.646 |
| methoxyacetic acid | -0.322 | -0.599 | -0.342 | -0.610 |
| 3-methylglutaric acid | -0.330 | -0.686 | -0.355 | -0.734 |
| 2-methylhexanedioic acid | -0.353 | -0.683 | -0.268 | -0.662 |
| 2-pentenoic acid | -0.106 | -0.603 | 0.120 | -0.451 |
| penta-2,4-dienoic acid | -0.114 | -0.644 | 0.051 | -0.539 |
| 4-chlorobutanoic acid | -0.186 | -0.720 | -0.090 | -0.675 |
| ethoxyacetic acid | -0.405 | -0.579 | -0.305 | -0.468 |
| (ethylthio)acetic acid | -0.370 | -0.640 | -0.262 | -0.530 |
| 5-methylhexanoic acid | 0.011 | -0.558 | 0.432 | -0.272 |
| 2,6-dimethylbenzoic acid | -0.106 | -0.721 | 0.221 | -0.490 |
| tetrachlorophthalic acid | -0.834 | -1.372 | -0.827 | -1.406 |
| 1h-pyrrole-2-carboxylic acid | -0.378 | -0.745 | -0.472 | -0.781 |
| 3-pyridinesulfonic acid | 0.134 | -0.611 | 0.211 | -0.601 |
| 1-cyclohexenylcarboxylic acid | -0.049 | -0.568 | 0.322 | -0.318 |
| tridecanoic acid | 0.264 | -0.410 | 0.936 | 0.056 |
| 5-nitro-2-furoic acid | -0.572 | -1.042 | -0.727 | -1.155 |
| isocaproic acid | -0.027 | -0.594 | 0.303 | -0.361 |
| nonadecanoic acid | 0.428 | -0.312 | 1.190 | 0.234 |
| 5-aminovaleric acid | 0.662 | 0.427 | 0.473 | -0.134 |
| 2,2-dimethylglutaric acid | -0.332 | -0.723 | -0.283 | -0.725 |
| dodecanedioic acid | -0.133 | -0.676 | 0.285 | -0.464 |
| cyclohexanepropanoic acid | 0.047 | -0.536 | 0.525 | -0.208 |
| 5-methyl-5-cyclohexylbarbituric acid | -0.142 | -0.565 | 0.031 | -0.436 |
| anthracene-9-carboxylic acid | -0.073 | -0.726 | 0.314 | -0.453 |
| 3-methyl-2-oxobutanoic acid | -0.396 | -0.566 | -0.403 | -0.431 |
| hex-2-ynoic acid | -0.090 | -0.716 | 0.148 | -0.572 |
| indole-3-carboxylic acid | -0.569 | -0.794 | -0.474 | -0.738 |
| 3,3-dimethyl-2-oxobutanoic acid | -0.368 | -0.561 | -0.273 | -0.380 |
| tetradecanedioic acid | -0.101 | -0.668 | 0.363 | -0.422 |
| p-methylphenoxyacetic acid | -0.441 | -0.745 | -0.259 | -0.582 |
| methylphosphonic acid | -0.281 | -0.643 | -0.608 | -1.021 |
| 8-aminocaprylic acid | -0.132 | -0.345 | 0.158 | -0.233 |
| pentadecanoic acid | 0.330 | -0.391 | 1.002 | 0.099 |
| tert-butylacetic acid | 0.002 | -0.568 | 0.359 | -0.328 |
| 4-ethenylbenzoic acid | -0.113 | -0.673 | 0.201 | -0.455 |
| 2,3,4-trimethylbenzoic acid | -0.059 | -0.591 | 0.381 | -0.297 |
| 3,4,5-trimethylbenzoic acid | -0.009 | -0.593 | 0.386 | -0.313 |
| 2-tert-butylbenzoic acid | -0.074 | -0.676 | 0.364 | -0.379 |
| 3-hydroxy-2-oxopropionic- acid | -0.404 | -0.761 | -0.777 | -0.981 |
| 5-chloropentanoic acid | -0.146 | -0.685 | 0.028 | -0.575 |
| 6-heptenoic acid | -0.066 | -0.618 | 0.270 | -0.379 |
| cyclopentylacetic acid | -0.012 | -0.572 | 0.361 | -0.314 |
| 3,5-dimethoxybenzoic acid | -0.179 | -0.677 | 0.138 | -0.485 |
| 2,6-naphthalenedicarboxylic acid | -0.369 | -0.926 | -0.208 | -0.889 |
| 2,2-dimethylvaleric acid | 0.077 | -0.537 | 0.466 | -0.252 |
| 2-hexenoic acid | -0.035 | -0.574 | 0.285 | -0.355 |
| 6-hydroxycaproic acid | -0.173 | -0.503 | 0.016 | -0.402 |
| 4-aminobenzeneacetic acid | -0.179 | -0.600 | 0.013 | -0.511 |
| 2,6-dimethoxybenzoic acid | -0.211 | -0.583 | -0.079 | -0.472 |
| trifluoromethanesulfonic acid | -2.066 | -2.369 | -2.026 | -2.309 |
| 2,3-dimethoxybenzoic acid | -0.136 | -0.391 | 0.187 | -0.076 |
| phenylphosphonic acid | -0.506 | -0.969 | -0.513 | -1.068 |
| 5-hexenoic acid | -0.102 | -0.638 | 0.176 | -0.447 |
| dehydro(3)abietic acid | 0.151 | -0.505 | 0.806 | -0.046 |
| cyclopropanecarboxylic acid | -0.109 | -0.621 | 0.014 | -0.548 |
| phenylphosphinic acid | -0.074 | -0.480 | 0.110 | -0.372 |
| m-methoxyphenylacetic acid | -0.171 | -0.675 | 0.069 | -0.517 |
| 4-phenylbutyric acid | -0.077 | -0.651 | 0.292 | -0.396 |
| 2-nonynoic acid | 0.042 | -0.638 | 0.462 | -0.354 |
| m-cyanobenzoic acid | -0.139 | -0.726 | 0.015 | -0.619 |
| 5-methyl-2-furancarboxylic acid | -0.223 | -0.685 | -0.110 | -0.603 |
| 4-amino-3,5,6-trichloropyridine-2-carboxylic acid | 0.159 | -0.337 | 0.330 | -0.111 |
| palustric acid | 0.187 | -0.439 | 0.863 | 0.041 |
| 2-propenylesterbutanoic acid | 0.137 | -0.244 | 0.640 | 0.242 |
| 2-bromo-2-methylpropanoic acid | -0.486 | -0.924 | -0.306 | -0.788 |
| 3,3'-selenobispropanoic acid | -0.412 | -0.861 | -0.403 | -0.928 |
| pentamethylbenzoic acid | 0.037 | -0.613 | 0.501 | -0.302 |
| 4-phenyl-3-butenoic acid | -0.168 | -0.711 | 0.139 | -0.498 |
| 3-hydroxy-4-aminobenzoic acid | -0.541 | -0.852 | -0.621 | -0.960 |
| dodecaneperoxoic acid | 0.485 | -0.049 | 1.079 | 0.529 |
| oxiniacic acid | -0.107 | -0.514 | -0.098 | -0.525 |
| 2,3,4,6-tetramethylbenzoic acid | 0.021 | -0.613 | 0.457 | -0.313 |
| eicosanedioic acid | 0.096 | -0.575 | 0.708 | -0.197 |
| 2,3,5-trimethylbenzoic acid | -0.027 | -0.593 | 0.378 | -0.301 |
| 2-(1-methylethyl)-benzoic acid | -0.071 | -0.643 | 0.370 | -0.344 |
| 4-propylbenzoic acid | -0.024 | -0.586 | 0.413 | -0.283 |
| urb acid | 0.457 | -0.191 | 0.991 | 0.303 |
| pyrophosphoric acid | -1.519 | -1.752 | -1.958 | -2.322 |
| 4-amino-m-toluic acid | -0.176 | -0.606 | -0.012 | -0.486 |
| 2-azetidinecarboxylic acid | -0.121 | -0.403 | -0.036 | -0.262 |
| 2,3,6-trimethylbenzoic acid | -0.059 | -0.686 | 0.322 | -0.425 |
| 2,3,4,5-tetramethylbenzoic acid | -0.002 | -0.555 | 0.477 | -0.228 |
| 2,3,5,6-tetramethylbenzoic acid | -0.040 | -0.660 | 0.358 | -0.380 |
| o-diselenane-3,6-dicarboxylic acid | -0.460 | -0.976 | -0.427 | -1.052 |
| 2,5-dimethoxybenzoic acid | -0.221 | -0.427 | 0.097 | -0.113 |
| 1-hydroxyethylidene-1,1-diphosphonic acid | -1.005 | -1.189 | -1.399 | -1.583 |
| 4-oxo-pent-2-enoic acid | -0.115 | -0.649 | -0.020 | -0.641 |
| 2-methyl-octanoic acid | 0.136 | -0.494 | 0.659 | -0.125 |
| 4-hydroxymethyl-benzoic- acid | -0.167 | -0.649 | -0.011 | -0.620 |
| 5-oxohexanoic acid | -0.292 | -0.523 | -0.112 | -0.411 |
| 6-amino-3-pyridinecarboxylic acid | -0.075 | -0.478 | 0.000 | -0.467 |
| 3-isoxazolecarboxylic acid | -0.335 | -0.797 | -0.417 | -0.846 |
| 2,5-furandicarboxylic acid | -0.672 | -1.113 | -0.842 | -1.333 |
| dimethylphosphinic acid | -0.080 | -0.317 | -0.049 | -0.351 |
| cyclopentanecarboxylic acid | -0.042 | -0.585 | 0.290 | -0.360 |
| 3,6-dichlorosalicylic acid | -0.879 | -0.897 | -0.767 | -0.725 |
| 5-oxooctanoic- acid | -0.296 | -0.529 | -0.003 | -0.296 |
| 2-ethyl-2-hydroxybutyric acid | -0.009 | -0.549 | 0.340 | -0.341 |
| cyclobutanecarboxylic acid | -0.093 | -0.627 | 0.137 | -0.475 |
| 3-5-diethylbenzoic acid | 0.039 | -0.575 | 0.511 | -0.241 |
| methylphosphinic acid | -0.190 | -0.603 | -0.225 | -0.741 |
| ethyl-boronic acid | -0.086 | -0.568 | -0.031 | -0.548 |
| 2-methylhexanoic acid | 0.049 | -0.547 | 0.469 | -0.250 |
| propylphosphonic acid | -0.331 | -0.751 | -0.356 | -0.876 |
| 6-chloropicolinic acid | -0.104 | -0.420 | -0.068 | -0.235 |
| 2,2-bis(hydroxymethyl)propanoic acid | -0.314 | -0.521 | -0.237 | -0.510 |
| 3-cyclohexenylcarboxylic acid | -0.131 | -0.648 | 0.198 | -0.433 |
| cyclohexaneacetic- acid | 0.008 | -0.560 | 0.454 | -0.260 |
| sulfamic acid | -1.184 | -0.857 | -2.114 | -1.752 |
| 3-(1-methylethyl)-benzoic acid | -0.015 | -0.604 | 0.431 | -0.305 |
| isopimaric acid | 0.169 | -0.474 | 0.860 | 0.001 |
| 2-hydroxyhexanoic acid | -0.098 | -0.570 | 0.209 | -0.380 |
| 4-pentynoic acid | -0.253 | -0.751 | -0.230 | -0.743 |
| 1,2-ethanediylbisphosphonic acid | -0.353 | -0.821 | -0.581 | -1.079 |
| 2,2'-selenobisacetic acid | -0.497 | -0.955 | -0.745 | -1.238 |
| hypophosphorous acid | -0.103 | -0.399 | -0.381 | -0.705 |
| (phenylmethyl)selenopropanoic acid | -0.186 | -0.763 | 0.129 | -0.524 |
| phenylseleno acid | -0.049 | -0.509 | 0.134 | -0.362 |
| 3,3'-diselenobispropanoic acid | -0.521 | -0.920 | -0.462 | -0.923 |
| octahydro-pentalene-1-carboxylic acid | -0.002 | -0.539 | 0.461 | -0.229 |
| 3-tert-butylbenzoic acid | -0.004 | -0.610 | 0.448 | -0.296 |
| perchloric acid | -3.182 | -2.774 | -3.219 | -2.760 |
| phosphoric acid | -1.302 | -1.370 | -2.018 | -2.113 |
| nitric acid | -2.134 | -1.926 | -2.373 | -2.057 |
| arsenic acid | -1.081 | -1.259 | -1.726 | -1.898 |
| nitrous acid | -1.235 | -1.290 | -1.368 | -1.322 |
| nitrosylsulfuric acid | -0.648 | -1.051 | -1.185 | -1.469 |
| hydrazoic acid | -1.008 | -0.983 | -0.520 | -0.598 |
| selenious acid | -0.433 | -0.787 | -0.957 | -1.282 |
| selenic acid | -2.153 | -2.190 | -2.753 | -2.758 |
| fluorosulfuric acid | -2.880 | -2.895 | -2.945 | -2.927 |
| hypochlorous acid | -1.130 | -1.364 | -1.272 | -1.432 |
| chlorosulfonic acid | -2.663 | -2.757 | -2.731 | -2.776 |
| boric acid | -0.200 | -0.533 | -0.601 | -0.997 |
| parasorbic acid | -0.274 | -0.427 | -0.011 | -0.150 |
| triphosphoric acid | -1.363 | -1.810 | -1.788 | -2.241 |
| 4-methylaminobenzoic acid | -0.107 | -0.576 | 0.037 | -0.448 |
| hexanebis(thioic) acid | 0.032 | -0.374 | 0.260 | -0.049 |
| methylboronic acid | -0.062 | -0.547 | -0.116 | -0.666 |
| hypobromous acid | -0.884 | -1.209 | -0.859 | -1.169 |
| phosphorous acid | -0.502 | -0.835 | -1.001 | -1.355 |
| acetoxyacetic acid | -0.210 | -0.707 | -0.248 | -0.782 |
| peroxydecanoic acid | 0.300 | -0.158 | 0.765 | 0.338 |
| 9-decenoic- acid | 0.042 | -0.538 | 0.566 | -0.171 |
| (e)-2-nonenoic- acid | 0.102 | -0.509 | 0.583 | -0.167 |
| p-methacryloyloxybenzoic acid | -0.143 | -0.650 | 0.215 | -0.422 |
| homocubane-4-carboxylic acid | -0.069 | -0.560 | 0.348 | -0.281 |
| ethylphosphonic acid | -0.324 | -0.723 | -0.488 | -0.948 |
| 3,5-di-tert-butylbenzoic acid | 0.149 | -0.512 | 0.724 | -0.109 |
| tetrahydro-2-furancarboxylic acid | -0.394 | -0.501 | -0.284 | -0.340 |
| propylboronic acid | -0.022 | -0.531 | 0.175 | -0.411 |
| tetrahydro-2,5-selenophenedicarboxylic acid | -0.395 | -0.918 | -0.412 | -1.054 |
| (e)-2-heptenoic- acid | 0.005 | -0.554 | 0.381 | -0.288 |
| dilactic acid | -0.052 | -0.654 | 0.047 | -0.641 |
| tetradecaneperoxoic acid | 0.599 | 0.004 | 1.286 | 0.623 |
| 4-butylbenzoic acid | 0.039 | -0.559 | 0.540 | -0.217 |
| 1-aminocyclopropanecarboxylic acid | -0.063 | -0.402 | -0.043 | -0.306 |
| 2,2'-selenobispropanoic acid | -0.354 | -0.863 | -0.271 | -0.925 |
| 2,2'-diselenobis-2-methylpropionic acid | -0.279 | -0.847 | -0.023 | -0.763 |
| acryloxypropionic acid | -0.255 | -0.647 | -0.128 | -0.592 |
| 2-methylcyclopropanecarboxylic acid | -0.079 | -0.593 | 0.170 | -0.433 |
| p-nitrobenzylselenopropionic acid | -0.175 | -0.742 | 0.021 | -0.592 |
| zz-3-methyl-4-cyclohexene-1,2-dicarboxylic acid | 0.192 | -0.307 | 0.424 | -0.009 |
| peroxytridecanoic acid | 0.447 | -0.068 | 1.011 | 0.489 |
| p-methylbenzylselenopropionic acid | -0.152 | -0.724 | 0.266 | -0.435 |
| p-bromobenzylselenopropionic acid | -0.197 | -0.766 | 0.191 | -0.475 |
| dodecylbenzenesulfonic- acid | -0.181 | -0.905 | 0.413 | -0.496 |
| m-fluorobenzylselenopropionic acid | -0.193 | -0.758 | 0.171 | -0.506 |
| p-fluorobenzylselenopropionic acid | -0.187 | -0.755 | 0.186 | -0.497 |
| m-chlorobenzylselenopropionic acid | -0.201 | -0.779 | 0.107 | -0.537 |
| p-chlorobenzylselenopropionic acid | -0.118 | -0.730 | 0.252 | -0.469 |
| n-butyl-n-(2-propynyl)-s-ethylcarbamothioic acid | 0.250 | -0.217 | 0.818 | 0.308 |
| cyanosulfoxylic acid | -0.653 | -1.153 | -0.673 | -1.151 |
| 11-dodecenoic- acid | 0.133 | -0.485 | 0.719 | -0.067 |
| trilactic acid | -0.207 | -0.648 | -0.047 | -0.553 |
| 2-acetylbutanedioic acid | -0.331 | -0.669 | -0.539 | -0.865 |
| 4-carbamoylbenzoic acid | -0.096 | -0.550 | -0.076 | -0.571 |
| carbonic acid | -1.277 | -1.407 | -1.725 | -1.857 |
| nitroacetic acid | -0.853 | -1.040 | -1.277 | -1.298 |
| peroxoformic acid | -0.764 | -0.856 | -1.232 | -1.042 |
| (+)-(1s,2r)-2-heptylcyclopropanecarboxylic acid | 0.208 | -0.435 | 0.790 | -0.029 |
| (-)-(1r,2s)-2-heptylcyclopropanecarboxylic acid | 0.185 | -0.446 | 0.772 | -0.038 |
| (4r)-4-methylnonanoic acid | 0.132 | -0.488 | 0.683 | -0.102 |
| (4r)-4-methyloctanoic acid | 0.094 | -0.516 | 0.582 | -0.165 |
| (4s)-4-methylnonanoic acid | 0.145 | -0.490 | 0.696 | -0.104 |
| (4s)-4-methyloctanoic acid | 0.105 | -0.521 | 0.579 | -0.175 |
| (r)-(+)-4-ethyloctanoic acid | 0.130 | -0.492 | 0.632 | -0.118 |
| (r)-(+)-4-methylhexanoic acid | 0.007 | -0.574 | 0.398 | -0.298 |
| (r)-3-hydroxy-3-methylhexanoic acid | -0.065 | -0.463 | 0.311 | -0.214 |
| (s)-(-)-4-ethyloctanoic acid | 0.139 | -0.501 | 0.643 | -0.133 |
| (s)-(-)-4-methylhexanoic acid | 0.022 | -0.570 | 0.398 | -0.300 |
| (s)-3-hydroxy-3-methylhexanoic acid | -0.048 | -0.472 | 0.301 | -0.236 |
| 1,3-dimethylbarbituric acid | -0.113 | -0.413 | 0.020 | -0.197 |
| 5,5-dibromo-barbituric acid | -0.661 | -0.946 | -0.860 | -1.060 |
| 5,5-dichlorobarbituric acid | -0.670 | -0.973 | -0.940 | -1.163 |
| cyclopenteneacetic acid | -0.188 | -0.644 | 0.118 | -0.428 |
| p-coumaric acid | -0.527 | -0.927 | -0.430 | -0.919 |
| aspirin | -0.196 | -0.654 | 0.032 | -0.523 |
| glycine | 0.475 | 0.442 | 0.051 | -0.292 |

**Table S2.** The substituent effects on the intermolecular interaction (kcal/mol) and charge transfer (me^-^) of para substituted analogues of benzoic acid with benzamide and urea. Here σ_p_ stands for Hammett constant, ΔE(U) and ΔE(B) denote intermolecular interaction energy of the pair comprising urea or benzoic acid, Q_1->2_, Q_2->1_ denote amount of charge transfer from amide to acid and vice versa, while the last two column represent values of the total charge transfer in the given pair.

| **substituent** | **σ_p_** [43]. | **ΔE(U)** | **ΔE(B)** | **Q(U)_1->2_** | **Q(U)_2->1_** | **Q(B)_1->2_** | **Q(B)_2->1_** | **Q(U)** | **Q(B)** |
| --- | --- | --- | --- | --- | --- | --- | --- | --- | --- |
| H | 0.00 | -18.0 | -17.7 | 7.7 | 3.1 | 6.9 | 2.6 | 10.8 | 9.5 |
| BF2 | 0.48 | -18.6 | -18.2 | 8.3 | 3.0 | 7.5 | 2.3 | 11.3 | 9.7 |
| Br | 0.23 | -18.4 | -18.0 | 8.1 | 3.0 | 7.2 | 2.4 | 11.1 | 9.6 |
| SiBr3 | 0.57 | -18.7 | -18.3 | 8.4 | 2.9 | 7.6 | 2.2 | 11.4 | 9.8 |
| Cl | 0.23 | -18.4 | -18.0 | 8.1 | 3.0 | 7.2 | 2.4 | 11.1 | 9.6 |
| SO2Cl | 1.11 | -19.5 | -19.0 | 9.2 | 2.8 | 8.3 | 2.0 | 12.0 | 10.4 |
| SCl | 0.48 | -18.8 | -18.4 | 8.5 | 2.9 | 7.7 | 2.2 | 11.4 | 9.8 |
| ICl2 | 1.11 | -19.5 | -19.0 | 9.1 | 2.8 | 8.3 | 2.1 | 11.9 | 10.3 |
| POCl2 | 0.90 | -19.2 | -18.8 | 8.9 | 2.9 | 8.0 | 2.2 | 11.8 | 10.2 |
| PCl2 | 0.61 | -18.8 | -18.4 | 8.5 | 2.9 | 7.6 | 2.2 | 11.5 | 9.8 |
| PSCl2 | 0.80 | -19.1 | -18.7 | 8.8 | 2.9 | 7.9 | 2.2 | 11.7 | 10.1 |
| SiCl3 | 0.56 | -18.7 | -18.3 | 8.5 | 2.9 | 7.6 | 2.3 | 11.4 | 9.9 |
| F | 0.06 | -18.3 | -17.9 | 7.9 | 3.1 | 7.1 | 2.5 | 11.0 | 9.6 |
| SOF | 0.83 | -19.1 | -18.7 | 8.8 | 2.9 | 7.9 | 2.2 | 11.7 | 10.0 |
| SO2F | 0.91 | -19.5 | -19.0 | 9.2 | 2.8 | 8.3 | 2.0 | 12.0 | 10.4 |
| IF2 | 0.83 | -19.0 | -18.5 | 8.6 | 2.9 | 7.8 | 2.2 | 11.5 | 10.0 |
| POF2 | 0.89 | -19.3 | -18.8 | 9.0 | 2.8 | 8.1 | 2.2 | 11.8 | 10.3 |
| PF2 | 0.59 | -18.7 | -18.4 | 8.5 | 2.9 | 7.6 | 2.2 | 11.4 | 9.8 |
| SF3 | 0.80 | -19.3 | -18.8 | 9.0 | 2.8 | 8.1 | 2.0 | 11.8 | 10.1 |
| SiF3 | 0.69 | -18.9 | -18.5 | 8.6 | 2.9 | 7.7 | 2.1 | 11.5 | 9.9 |
| IF4 | 1.15 | -19.6 | -19.1 | 9.2 | 2.7 | 8.4 | 2.0 | 12.0 | 10.3 |
| PF4 | 0.80 | -19.2 | -18.8 | 8.9 | 2.8 | 8.1 | 2.1 | 11.8 | 10.2 |
| SF5 | 0.68 | -19.2 | -18.8 | 9.0 | 2.9 | 8.1 | 2.1 | 11.8 | 10.2 |
| I | 0.18 | -18.4 | -18.0 | 8.1 | 3.0 | 7.2 | 2.4 | 11.1 | 9.7 |
| IO | 0.62 | -19.1 | -18.6 | 8.7 | 2.9 | 7.9 | 2.2 | 11.6 | 10.1 |
| IO2 | 0.78 | -19.3 | -18.8 | 8.9 | 2.8 | 8.1 | 2.2 | 11.7 | 10.3 |
| NO | 0.91 | -19.0 | -18.5 | 8.7 | 2.9 | 7.9 | 2.2 | 11.6 | 10.1 |
| NO2 | 0.78 | -19.2 | -18.7 | 8.9 | 2.8 | 8.1 | 2.1 | 11.7 | 10.2 |
| ONO2 | 0.70 | -18.7 | -18.3 | 8.4 | 3.0 | 7.5 | 2.3 | 11.4 | 9.8 |
| N3 | 0.08 | -18.2 | -17.9 | 7.9 | 3.1 | 7.1 | 2.4 | 11.0 | 9.4 |
| NHNO2 | 0.57 | -18.6 | -18.3 | 8.2 | 3.0 | 7.3 | 2.3 | 11.2 | 9.6 |
| OH | -0.37 | -17.7 | -17.5 | 7.5 | 3.2 | 6.6 | 2.7 | 10.7 | 9.3 |
| SH | 0.15 | -18.0 | -17.7 | 7.7 | 3.2 | 6.8 | 2.5 | 10.9 | 9.3 |
| B(OH)2 | 0.12 | -17.8 | -17.5 | 7.6 | 3.2 | 6.6 | 2.5 | 10.7 | 9.1 |
| NH2 | -0.66 | -17.4 | -17.3 | 7.1 | 3.4 | 6.1 | 2.8 | 10.5 | 8.9 |
| NHOH | -0.34 | -17.7 | -17.5 | 7.3 | 3.3 | 6.4 | 2.7 | 10.6 | 9.1 |
| SO2NH2 | 0.60 | -18.8 | -18.4 | 8.5 | 3.0 | 7.6 | 2.2 | 11.5 | 9.8 |
| PO(OH)2 | 0.42 | -18.6 | -18.2 | 8.3 | 3.0 | 7.3 | 2.3 | 11.3 | 9.6 |
| PH2 | 0.05 | -18.1 | -17.8 | 7.8 | 3.1 | 6.9 | 2.5 | 10.9 | 9.4 |
| NHNH2 | -0.55 | -17.5 | -17.3 | 7.2 | 3.4 | 6.3 | 2.8 | 10.6 | 9.0 |
| SiH3 | 0.10 | -18.1 | -17.8 | 7.9 | 3.1 | 7.0 | 2.5 | 11.0 | 9.5 |
| CBr3 | 0.29 | -18.6 | -18.2 | 8.3 | 3.0 | 7.4 | 2.2 | 11.4 | 9.6 |
| CClF2 | 0.46 | -18.7 | -18.3 | 8.5 | 3.0 | 7.5 | 2.2 | 11.4 | 9.7 |
| COCl | 0.61 | -19.1 | -18.6 | 8.8 | 2.9 | 7.9 | 2.1 | 11.7 | 10.0 |
| N=CCl2 | 0.13 | -18.2 | -17.9 | 8.0 | 3.1 | 7.0 | 2.4 | 11.1 | 9.4 |
| CCl3 | 0.46 | -18.6 | -18.2 | 8.4 | 3.0 | 7.4 | 2.2 | 11.4 | 9.6 |
| OCCl3 | 0.35 | -18.6 | -18.2 | 8.3 | 3.0 | 7.4 | 2.3 | 11.3 | 9.7 |
| COF | 0.70 | -19.0 | -18.6 | 8.8 | 2.9 | 7.9 | 2.1 | 11.7 | 9.9 |
| CF3 | 0.54 | -18.8 | -18.4 | 8.5 | 3.0 | 7.6 | 2.2 | 11.5 | 9.7 |
| N=NCF3 | 0.68 | -19.0 | -18.6 | 8.7 | 2.9 | 7.9 | 2.2 | 11.6 | 10.0 |
| OCF3 | 0.35 | -18.6 | -18.2 | 8.3 | 3.0 | 7.4 | 2.3 | 11.3 | 9.7 |
| SOCF3 | 0.69 | -19.0 | -18.5 | 8.7 | 2.9 | 7.8 | 2.2 | 11.6 | 9.9 |
| SeOCF3 | 0.83 | -19.0 | -18.6 | 8.7 | 2.9 | 7.8 | 2.2 | 11.6 | 10.0 |
| SO2CF3 | 0.96 | -19.5 | -18.9 | 9.2 | 2.8 | 8.3 | 2.0 | 12.0 | 10.3 |
| SeO2CF3 | 1.21 | -19.6 | -19.1 | 9.3 | 2.7 | 8.4 | 2.1 | 12.0 | 10.5 |
| OSO2CF3 | 0.53 | -18.8 | -18.4 | 8.5 | 3.0 | 7.6 | 2.3 | 11.4 | 9.8 |
| SCF3 | 0.50 | -18.7 | -18.3 | 8.5 | 3.0 | 7.5 | 2.2 | 11.4 | 9.7 |
| CN | 0.66 | -19.0 | -18.6 | 8.7 | 2.9 | 7.8 | 2.1 | 11.7 | 9.9 |
| N=C=O | 0.19 | -18.3 | -18.0 | 8.0 | 3.1 | 7.1 | 2.3 | 11.1 | 9.3 |
| OCN | 0.54 | -18.8 | -18.4 | 8.5 | 2.9 | 7.6 | 2.1 | 11.4 | 9.7 |
| SO2CN | 1.26 | -19.7 | -19.2 | 9.4 | 2.7 | 8.6 | 2.0 | 12.2 | 10.6 |
| N=C=S | 0.38 | -18.5 | -18.1 | 8.2 | 3.1 | 7.2 | 2.2 | 11.3 | 9.4 |
| SCN | 0.52 | -18.8 | -18.4 | 8.4 | 3.0 | 7.6 | 2.3 | 11.4 | 9.9 |
| SeCN | 0.66 | -18.8 | -18.4 | 8.4 | 3.0 | 7.6 | 2.4 | 11.4 | 9.9 |
| C(NO2)3 | 0.82 | -19.5 | -19.0 | 9.3 | 2.9 | 8.5 | 2.1 | 12.2 | 10.5 |
| CHBr2 | 0.32 | -18.5 | -18.2 | 8.3 | 3.0 | 7.3 | 2.2 | 11.3 | 9.5 |
| CHCl2 | 0.32 | -18.5 | -18.1 | 8.3 | 3.0 | 7.3 | 2.2 | 11.3 | 9.5 |
| OCHCl2 | 0.26 | -18.6 | -18.2 | 8.2 | 3.0 | 7.3 | 2.3 | 11.2 | 9.6 |
| CHF2 | 0.32 | -18.6 | -18.2 | 8.3 | 3.0 | 7.4 | 2.2 | 11.3 | 9.5 |
| OCHF2 | 0.18 | -18.4 | -18.0 | 8.1 | 3.1 | 7.2 | 2.5 | 11.2 | 9.7 |
| SOCHF2 | 0.58 | -18.7 | -18.3 | 8.4 | 3.0 | 7.5 | 2.2 | 11.4 | 9.8 |
| SO2CHF2 | 0.86 | -19.2 | -18.7 | 9.0 | 2.9 | 8.1 | 2.1 | 11.8 | 10.2 |
| SCHF2 | 0.37 | -18.6 | -18.2 | 8.3 | 3.0 | 7.4 | 2.3 | 11.3 | 9.6 |
| CHI2 | 0.26 | -18.4 | -18.0 | 8.2 | 3.1 | 7.2 | 2.3 | 11.3 | 9.5 |
| NHCN | 0.06 | -18.4 | -18.1 | 8.0 | 3.1 | 7.1 | 2.5 | 11.1 | 9.6 |
| CHO | 0.42 | -18.8 | -18.4 | 8.4 | 3.0 | 7.5 | 2.2 | 11.4 | 9.7 |
| COOH | 0.45 | -18.6 | -18.2 | 8.3 | 3.0 | 7.4 | 2.2 | 11.3 | 9.6 |
| CH2Br | 0.14 | -18.3 | -17.9 | 8.0 | 3.1 | 7.1 | 2.4 | 11.1 | 9.4 |
| CH2Cl | 0.12 | -18.3 | -17.9 | 8.0 | 3.1 | 7.1 | 2.4 | 11.1 | 9.4 |
| OCH2Cl | 0.08 | -18.0 | -17.7 | 7.8 | 3.2 | 6.9 | 2.7 | 11.0 | 9.5 |
| CH2F | 0.11 | -18.3 | -17.9 | 8.0 | 3.1 | 7.0 | 2.3 | 11.1 | 9.4 |
| OCH2F | 0.02 | -18.0 | -17.7 | 7.8 | 3.2 | 6.8 | 2.6 | 11.0 | 9.5 |
| SCH2F | 0.20 | -18.2 | -17.8 | 7.9 | 3.1 | 7.0 | 2.3 | 11.0 | 9.4 |
| CH2I | 0.11 | -18.2 | -17.9 | 8.0 | 3.1 | 7.0 | 2.3 | 11.1 | 9.3 |
| NHCHO | 0.00 | -18.3 | -18.0 | 8.0 | 3.1 | 7.1 | 2.4 | 11.1 | 9.5 |
| CONH2 | 0.36 | -18.6 | -18.2 | 8.4 | 3.1 | 7.4 | 2.3 | 11.4 | 9.7 |
| CSNH2 | 0.30 | -18.3 | -18.0 | 8.2 | 3.0 | 7.2 | 2.3 | 11.2 | 9.5 |
| Me | -0.17 | -17.8 | -17.5 | 7.5 | 3.3 | 6.5 | 2.5 | 10.8 | 9.0 |
| SiMeCl2 | 0.39 | -18.4 | -18.0 | 8.2 | 3.1 | 7.4 | 2.4 | 11.3 | 9.8 |
| SiMeF2 | 0.23 | -18.4 | -18.1 | 8.1 | 3.0 | 7.3 | 2.3 | 11.2 | 9.6 |
| N(Me)NO2 | 0.61 | -18.6 | -18.2 | 8.2 | 3.0 | 7.2 | 2.3 | 11.3 | 9.6 |
| OMe | -0.27 | -17.6 | -17.4 | 7.4 | 3.3 | 6.5 | 2.8 | 10.7 | 9.2 |
| CH2OH | 0.00 | -17.8 | -17.6 | 7.5 | 3.3 | 6.5 | 2.5 | 10.8 | 9.0 |
| SOMe | 0.49 | -18.0 | -17.7 | 7.7 | 3.2 | 6.8 | 2.4 | 10.9 | 9.2 |
| S(O)OMe | 0.54 | -18.6 | -18.2 | 8.4 | 3.0 | 7.6 | 2.2 | 11.4 | 9.8 |
| SO2Me | 0.72 | -18.9 | -18.5 | 8.7 | 2.9 | 7.8 | 2.2 | 11.6 | 10.0 |
| SSO2Me | 0.54 | -18.5 | -18.1 | 8.3 | 3.0 | 7.4 | 2.3 | 11.3 | 9.6 |
| OSO2Me | 0.36 | -18.3 | -18.0 | 8.0 | 3.1 | 7.1 | 2.4 | 11.1 | 9.4 |
| SMe | 0.00 | -17.8 | -17.6 | 7.6 | 3.2 | 6.6 | 2.4 | 10.8 | 9.1 |
| SSMe | 0.13 | -18.0 | -17.7 | 7.7 | 3.2 | 6.8 | 2.4 | 10.9 | 9.2 |
| SeMe | 0.00 | -17.8 | -17.7 | 7.6 | 3.2 | 6.7 | 2.3 | 10.9 | 9.0 |
| NHMe | -0.70 | -17.3 | -17.2 | 7.0 | 3.5 | 6.0 | 3.0 | 10.5 | 9.0 |
| CH2NH2 | -0.11 | -18.0 | -17.7 | 7.7 | 3.2 | 6.7 | 2.4 | 10.9 | 9.1 |
| COOMe | 0.45 | -18.4 | -18.1 | 8.2 | 3.1 | 7.3 | 2.3 | 11.3 | 9.6 |
| NHEt | -0.61 | -17.3 | -17.1 | 7.0 | 3.5 | 6.0 | 2.9 | 10.5 | 8.9 |
| N(Me)2 | -0.83 | -17.2 | -17.1 | 6.9 | 3.5 | 6.0 | 2.8 | 10.4 | 8.7 |
| CH(Me)Et | -0.12 | -17.7 | -17.5 | 7.6 | 3.3 | 6.6 | 2.5 | 10.9 | 9.1 |
| N(Et)2 | -0.72 | -17.2 | -17.1 | 6.9 | 3.5 | 5.9 | 2.8 | 10.4 | 8.7 |
| N(C3H7)2 | -0.93 | -17.2 | -17.1 | 6.9 | 3.5 | 5.9 | 2.8 | 10.4 | 8.6 |
| SiMe(Ph)2 | 0.13 | -18.0 | -17.7 | 7.8 | 3.3 | 6.7 | 2.6 | 11.0 | 9.3 |
